# Specific Metabolome Profile of Exhaled Breath Condensate in Patients with Shock and Respiratory Failure: A Pilot Study

**DOI:** 10.3390/metabo6030026

**Published:** 2016-09-01

**Authors:** Brice Fermier, Hélène Blasco, Emmanuel Godat, Cinzia Bocca, Joseph Moënne-Loccoz, Patrick Emond, Christian R. Andres, Marc Laffon, Martine Ferrandière

**Affiliations:** 1 Department of Anesthesiology and Intensive Care, CHRU Tours Bretonneau, 2 boulevard Tonnellé, 37044 Tours cedex 9, France; brice.fermier@gmail.com (B.F.); emmanuel.godat@gmail.com (E.G.); joseph.moenne-loccoz@univ-tours.fr (J.M-L.); marc.laffon@univ-tours.fr (M.L.); martine.ferrandiere@univ-tours.fr (M.F.); 2 Laboratoire de Biochimie et Biologie Moléculaire, CHRU Bretonneau, 2, boulevard Tonnellé, 37044 Tours cedex 9, France; christian.andres@univ-tours.fr; 3 INSERM U930, équipe Neurogenetics and Neurometabolomics, Université François Rabelais, 10 bd Tonnellé, 37000 Tours, France; patrick.emond@univ-tours.fr; 4 PPF, Université François Rabelais, 10 bd tonnellé, 37000 Tours, France; cinzia.bocca@gmail.com

**Keywords:** metabolomics, mass spectrometry, exhaled breath condensate, biomarkers, shock

## Abstract

Background: Shock includes different pathophysiological mechanisms not fully understood and remains a challenge to manage. Exhaled breath condensate (EBC) may contain relevant biomarkers that could help us make an early diagnosis or better understand the metabolic perturbations resulting from this pathological situation. Objective: we aimed to establish the metabolomics signature of EBC from patients in shock with acute respiratory failure in a pilot study. Material and methods: We explored the metabolic signature of EBC in 12 patients with shock compared to 14 controls using LC-HRMS. We used a non-targeted approach, and we performed a multivariate analysis based on Orthogonal Partial Least Square-Discriminant Analysis (OPLS-DA) to differentiate between the two groups of patients. Results: We optimized the procedure of EBC collection and LC-HRMS detected more than 1000 ions in this fluid. The optimization of multivariate models led to an excellent model of differentiation for both groups (Q2 > 0.4) after inclusion of only 6 ions. Discussion and conclusion: We validated the procedure of EBC collection and we showed that the metabolome profile of EBC may be relevant in characterizing patients with shock. We performed well in distinguishing these patients from controls, and the identification of relevant compounds may be promising for ICC patients.

## 1. Introduction

Shock is a challenge to manage in an Intensive Care Unit (ICU). The clinicians have to detect the symptoms early and adapt their management strategies in order to limit high morbidity and multiple system illness such as renal and respiratory dysfunctions [1]. Pathogenesis is related to molecular activation pathways, including dysregulated inflammatory responses [2,3]. Although several cytokines and soluble antigens have been described in bronco-alveolar lavage (BAL) [4,5], the pathophysiology of the deleterious mechanisms remains unknown. The mechanisms, especially the pulmonary response, and the role of immune cells in the shock have to be further explored. The metabolic impact of inflammation has been widely reported in various diseases such as cancer or neurodegenerative disorders [6,7]. Interestingly, some authors reported a relation between lipid peroxidation products in the breath of ventilated ICU patients and inflammatory markers in BAL [5].

Metabolomics refers to an emergent powerful strategy that provides metabolic patterns from a combination of small molecules. The analytical methods detect metabolites across a large spectrum of concentrations, polarity and masses [8,9,10,11]. The metabolome may reflect the evolution of physiological states in response to environmental variations or aggressions such as microbial infections. These approaches enable the semi-quantification of metabolites up to 1500 daltons, including organic acids, lipids, amino acids, carbohydrates, peptides, vitamins, steroids, xenobiotics and many others. Among the different analytical methods available to perform metabolomics analysis, liquid chromatography coupled with high resolution mass spectrometry (LC-HRMS) is one of the most sensitive and reliable methods. The biological fluids usually explored are urine, plasma, cerebrospinal fluid, and cell homogenates. Metabolomics studies are based on two different approaches: a global profiling strategy (i.e., untargeted) and a targeted strategy. The untargeted method is suitable to evaluate a metabolome profile without any a priori knowledge on the metabolic disturbances involved in the pathological condition.

Few data are available about metabolome exploration during shock, based on nuclear magnetic resonance or mass spectrometry [12,13,14,15]. Interestingly, some authors performed a metabolomics study in patients with severe septic shock and showed that early modifications of lipids and kynurenine plasma levels were associated with mortality [16]. We hypothesized that the exploration of a matrix directly extracted from the lung may be more informative to explore shock pathophysiology. To avoid invasive pulmonary samplings such as BAL, exhaled breath condensate (EBC) procedure has been developed and this fluid may be used for biomarkers research. This type of matrix may be particularly useful for diagnosis and longitudinal follow up of patients [17]. EBC is suitable for metabolomics analysis and may be a good approach to studying lung reactions during shock or lung diseases, based on standardized EBC sample collection [18,19]. Although some metabolomics works have been published on this fluid, based on LC-MS, GC-MS [20,21,22,23,24,25] or NMR [26,27], such an approach has never been applied to explore shock pathophysiology.

The aim of our study was to develop a protocol for EBC collection and to assess the metabolome profile from mechanically ventilated patients in shock with acute respiratory failure and, as controls, mechanically ventilated patients during anesthesia for programmed surgeries.

## 2. Results

### 2.1. Patients and Sampling

The characteristics of patients are summarized in Table 1. Patients (P) (*n* = 12) were admitted in the intensive care unit for the diagnosis of: septic shock (7 peritonitis, 2 pneumonia), anaphylactic shock (1), hemorrhagic shock (1), cardiogenic shock (1). Controls (*n* = 14) were admitted in the surgical unit for: vascular surgery (7) (femoral endarterectomy and femoral bypass), aortic surgery (6) and phlebotomy (1). Patients in both groups were receiving different medical therapies according to their surgical conditions and previous medical history.

During EBC sampling procedure, no complications were observed or recorded. Between 2 and 3 mL of condensate were collected in 20 min.

### 2.2. Metabolomics Analysis

**Figure 1:**
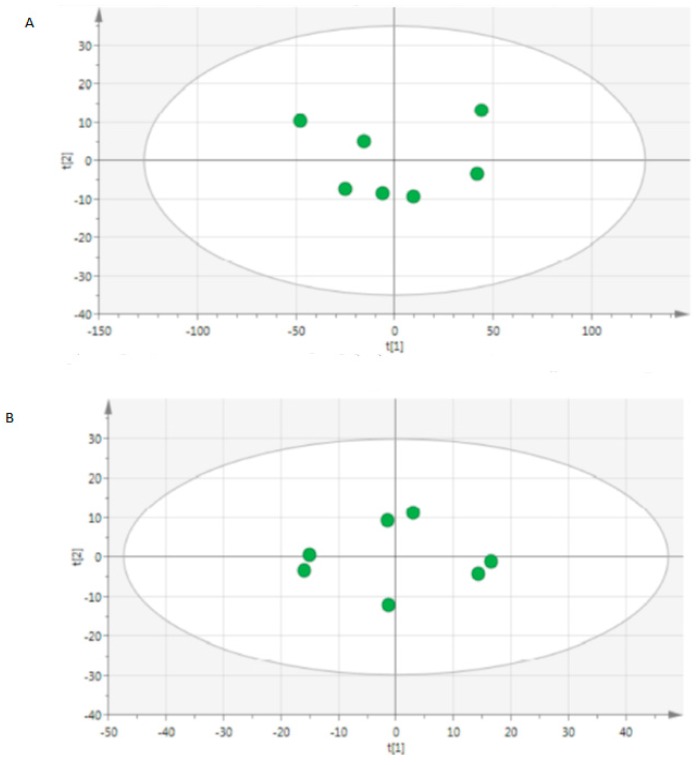
Score scatter plot of principal component analysis (PCA) from quality controls from (**A**) positive mode and (**B**) negative mode.

**Figure A1:**
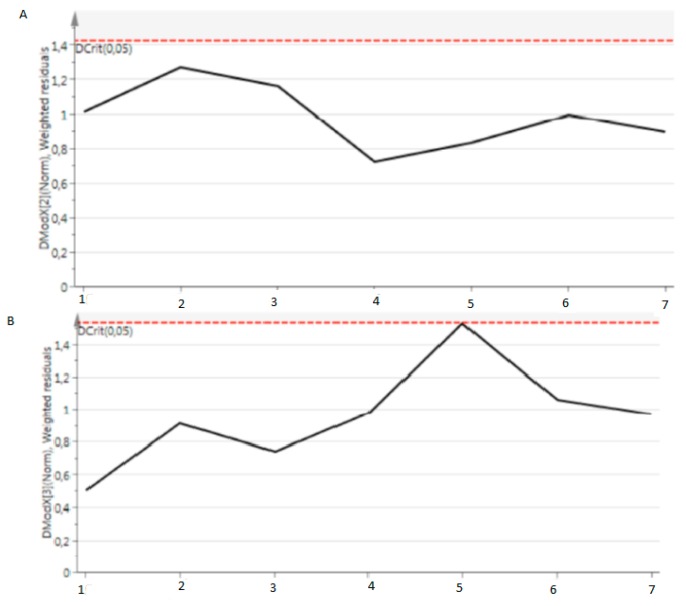
DModXPS data based metabolome obtained from quality controls. All quality controls are under the Dcrit (0.05) line.

A total of 10.972 peaks were detected in positive mode and 1.468 in negative mode. Each chromatogram was manually controlled to retain 1.762 peaks in positive mode and 545 peaks in negative mode. Secondary for each peak an isotopic enrichment was researched in order to confirm their correspondence with molecular ions. Finally 1.345 ions were kept in positive mode and 460 in negative mode. We validated the reproducibility of quality controls as shown in PCA plots (Figure 1 and Figure A1).

**Figure 2:**
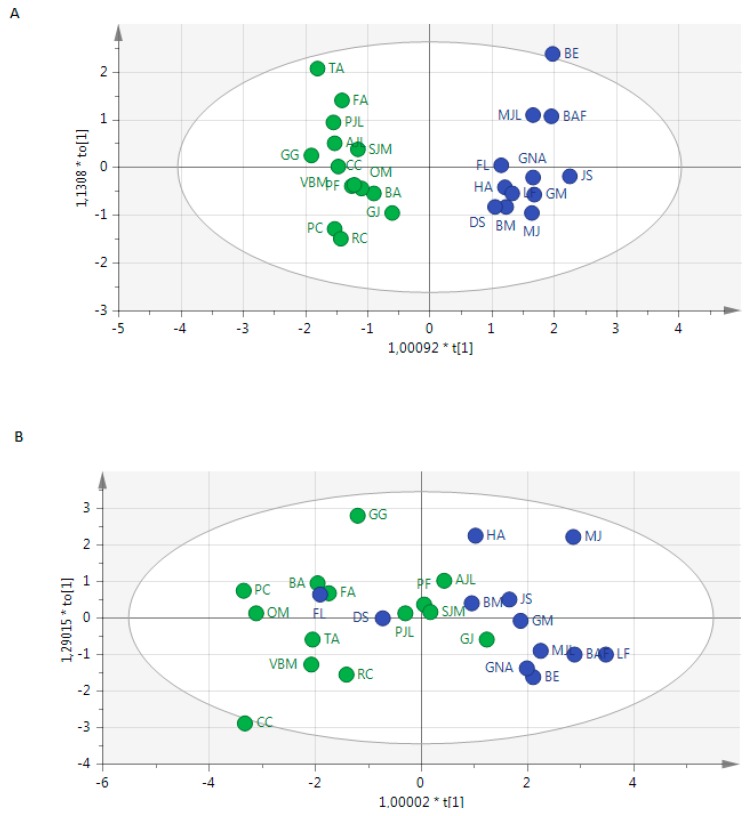
Score scatter plot of OPLS-DA from the metabolome of exhaled breath condensates showing the discrimination of patients (blue) and controls (green) using (**A**) positive mode and (**B**) negative mode.

Based on positive and negative ions data we showed a correct discrimination of 6 compounds between both groups (Figure 2). The PCA analysis of EBC metabolome within each group of patients revealed a correct homogeneity of profiles without any outlier in each group of patients (not shown).

**Figure 3:**
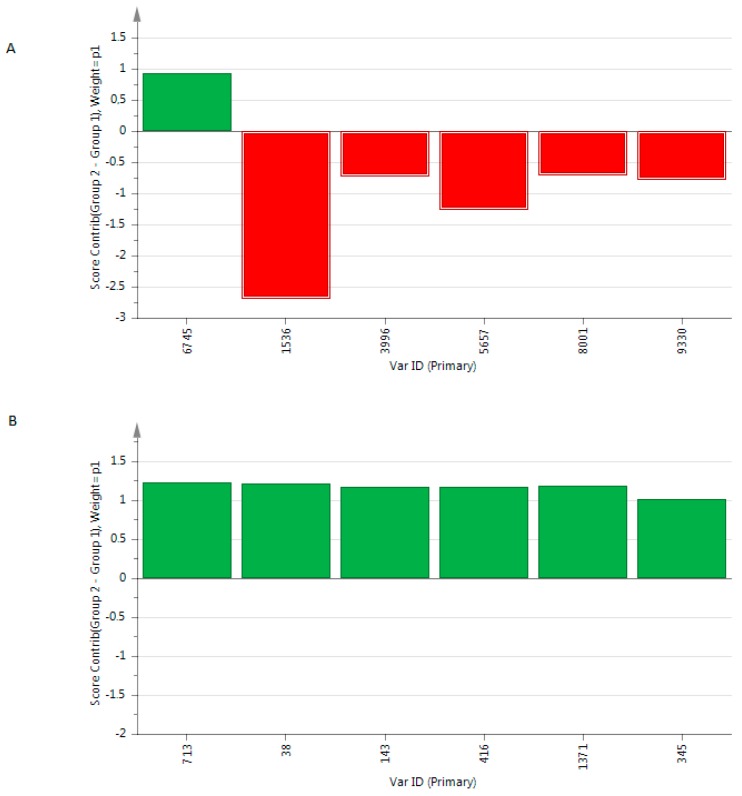
Contribution plot of the most relevant VIP included in the OPLS-DA models built from the metabolome of exhaled breath condensate based on (**A**) positive mode and (**B**) negative mode. Metabolites with positive score contributions are higher in patients than in controls.

We showed a R_2_X(cum) at 0.547 and 0.992, R_2_Y(cum) at 0.947 and 0.496 and a Q_2_ at 0.919 and 0.449, respectively. The metabolites with the highest variable importance in prediction (VIP) are presented in Figure 3.

Even if the design of this study is not suitable for diagnosis prediction, we noted that 100%, and 82.4% of patients were correctly classified using metabolome from positive and negative mode, respectively. Before optimization of the model and variables exclusion from positive data, and even after optimization from negative data, we noted that 3 patients (GJ, FL DS) were misclassified (Figure 2).

## 3. Material and Methods

### 3.1. Subjects

We recruited 26 patients, over 18 years old, including 7 pregnant women. We separated the patients into two groups: 12 patients admitted in the critical care unit (P) and 14 surgically programmed patients considered as the control group (C). The patients did not receive halogenated anesthesia. The local ethical committee approved the protocol and patients or person of trust gave the informed consent.

The patients (P) were admitted in the critical care unit for shock. They were sedated by continuous intravenous administration of midazolam, sufentanil and mechanically ventilated by oxygen-air. With respect to guidelines, monitoring was adapted to degree of illness and organ dysfunction for each patient. We did not include patients presenting with an acute respiratory distress syndrome (ARDS) with arterial PO2/FiO2 < 100.

The anesthetic procedures were standardized for controls (C) as they received intravenous general anesthesia with propofol and remifentanil, and they were mechanically ventilated by oxygen-air. Monitoring was initiated with ECG, pulse oximetry and non-invasive measurement of arterial blood pressure. In this group we did not include patients with chronic pulmonary disease or per-operative hemodynamic instability. EBC was collected during the first hour after intubation and before the start of surgery.

In both groups, the same fluid gas (air and oxygen) were delivered from hospital central gas with no external pollution, the same endotracheal tube and ventilator tubing. All collections were done in the morning in order to reduce the impact of circadian variability.

### 3.2. Exhaled Breath Condensate: Collection from Patients

EBC was collected from intubated patients with the Ecoscreen device (Viasys HealthCare, Hoechberg, Germany), which was positioned in the expiratory limb of the ventilator circuit.

During each measurement, all patients received a tidal volume of (8 mL/kg), FiO_2_at (0.35), a standardized respiratory rate of 15/min, and the already assigned PEEP (i.e., 0 or 8 cm H_2_O).

The EBC condenser cooled exhaled breath at condensation.

Collecting time for EBC was 20 min, producing approximately 1 mL condensate sample. Samples were aliquoted and stored at −80 °C until analysis in one batch.

### 3.3. UPLC-MS Analysis

UPLC-HRMS analysis was performed on a UPLC Ultimate 3000 system (Dionex), coupled to a Q-Exactive Mass Spectrometer (Thermo Scientific), operated in the positive (ESI+) and negative (ESI-) electrospray ionization modes (one run for each mode). Either in negative or in positive mode, the HESI source worked with a spray voltage of 3.5 kV, a capillary temperature of 380C-, a heater temperature of 350 h, sheath gas flow of 30 arbitrary units (40 neg), auxiliary gas flow of 15 (20 neg) arbitrary units and spare gas flow of 2 arbitrary units. During the full scan acquisition, range going from 66.70 to 1000.00 m/z, the instrument operated at 70.000 resolutions, with an Automatic Gain Control (AGC) target of 1 × 10^6^ and a maximum injection time (IT) of 120 ms. Chromatography was carried out using a Dionex UltiMate^®^ 3000 UHPLC equipped with a Phenomenex Kinetex 1.7 µm XB - C18, 150 mm × 2.10 mm, 100Å HPLC column kept at a temperature of 40 °C. A multi-step gradient (preceded by an equilibration time of *circa* 3 min), with a mobile phase A of 0.1% formic acid in water and a mobile phase B in acetonitrile (ACN) with 0.1% of formic acid, was utilized with a flow rate maintained at 0.3 mL/min during a runtime of 10.30 min for the negative mode and 20.50 for the positive one. Both gradients start with 100% of phase A and circa 1 min after, the percentage of organic phase were brought to 50% in 5.50 min before reaching the 100% at 18.00 min for the positive contrary to the negative that simply reach the 100% in 7.00 min. Circa 2 min after, the initial conditions of gradients were met again. The UHPLC autosampler temperature was set at 4 °C and the injection volume for each sample used was 20 µL.

The samples were randomized before injection leading to an injection order independent to the clinical status of subjects. Before each series of analysis, QC samples were injected to condition the column (2 injections of 2 QC samples). Furthermore, one QC sample was injected every 10 samples to monitor UPLC-HRMS reproducibility (7 QC per plate) [28].

### 3.4. Data Preprocessing

All the raw data were processed as one batch using the SIEVE^R^ software (Thermo Fisher Scientific) for the peak alignment and framing. This process results in a table of time-aligned detected features, with their retention time, m/z ratio and intensity in each sample (peak area).

As the SIEVE^R^ framing algorithm typically considers all areas higher than a cut-off value as a potential peak and we used a low cut of intensity value for sensitivity considerations, all the frames (peaks) were manually analyzed by checking the peak shape. Frames that were not associated to a classical peak shape were eliminated from the peak table.

We normalized each peak area to the total peak areas of each chromatogram and further performed statistical analysis on non-normalized and normalized data (each peak area to the total peak areas of each chromatogram). This normalization enabled us to prevent pre-analytical and analytical biases inherent to the differences of some respiratory parameters of patients.

### 3.5. Multivariate Data Analysis

The preprocessed data sets were linear-transformed and were used as an input for Simca P+ version 13.0 (Umetrics, Umea, Sweden). Data were mean centered and unit variance scaled to perform unsupervised principal components analysis (PCA) [29] to identify the similarity or the differences between sample profiles. Spectral variation was reduced to a series of principal components (PC), each representing correlated spectral changes and summarized in a score plot. PCs are new variables that are orthogonal to each other and explain progressively less variance in the data set. PCs were displayed in a two-dimensional score plot, allowing visualization of the distribution and grouping of the samples in the new variable space. Scores plots were visually inspected for grouping, trends or outliers in the data. If these outliers were also detected in the distance to model plot (DModX) which is based on the model residual variance, they were rejected from the model, and PCA model was rebuilt. Orthogonal partial least-squares discriminant analysis (OPLS-DA) was performed to evaluate variations in frames areas between both groups: variation in the measured data is partitioned into 2 blocks, one containing variations that correlates with the class identifier and the other containing variation that is orthogonal to the first block and thus does not contribute to discrimination between the defined groups [30]. We created a score plot to visualize the OPLS-DA model, and we characterized the contribution of metabolites to the separation of classes using the loading plot and the contribution plot. OPLS-DA was cross validated by withholding one-seventh of the samples in seven successive simulations such that each sample was omitted once in order to guard against over fitting, leading to OPLS-DA built from one so called “predictive” component and two or more orthogonal components. *Q*^2^ and *R^2^* were used to assess the robustness of the model. *R^2^* is defined as a fraction of the variance explained by a component. Cross validation of *R^2^* gives *Q^2^*, representing the fraction of total variation predicted by a component. Variable importance parameters (VIP) rank the compounds according to their contribution to the model. We first deleted VIP having a threshold of 1.0 from OPLS-DA. The quality of the models was described by the cumulative modeled variation in the X matrix *R*^2^X (cum), the cumulative modeled variation in the Y matrix *R*^2^Y (cum), and the cross validated predictive ability *Q*^2^ (cum) values. Models were rejected if they presented complete overlap of *Q*^2^ distributions (*Q*^2^ (cum) < 0) or low classification rates (*Q*^2^ (cum) < 0.05 and eigen values should be > 2). We considered a model robust enough if *Q^2^* > 40% and *R^2^* > 50%, but these cut off values have to be re-evaluated in biological conditions. The set of multiple models resulting from the cross validation is used to calculate jack-knifing uncertainty measures. We fixed the maximum number of iterations at 200 until convergence of the OPLS algorithm [31].

## 4. Discussion

This study is the first metabolomics study from exhaled breath condensate performed in shock conditions. Metabolomics is an emergent approach enabling exploration of metabolism without any a priori hypothesis on metabolic disturbance. NMR or GC-MS metabolomics technologies have different criteria for performance, diverse requirements, preparation time, expenses, specificities, and capabilities to identify molecules [32,33]. All have technical limits such as sensitivity and resolution. The high sensitivity of LC-HRMS makes it one of the most robust techniques available for metabolomics studies. The impressive technical progress portends better and faster characterisation of the metabolome. However, some practical, financial and technical problems remain limitations on the application of the metabolomics strategy, and hurdles must be overcome before any marker is ready for the clinic [34]. Phases for the development of diagnostic assays have been established (discovery, retrospective validation, prospective screening, and qualification phases) but few studies follow the whole line of these procedures [35].

We showed that there is a high reproducibility in metabolome analysis from exhaled breath condensate using LC-HRMS. This pilot study is promising as we were able to distinguish between the two populations. These findings open up the perspectives of pathophysiology analysis based on the identification of metabolites in these models to help in the characterization of endophenotypes.

It is crucial to include the medical therapies in the discussion of our findings. We suggest that the discriminant VIPs identified in this study are independent from medication because (1) different drugs have been administered within each group without any difference in the types of treatment between both groups; (2) there is no statistical difference (non-parametric univariate analysis, not shown) of these metabolites levels between both groups; and (3) the respiratory elimination of drug metabolites and their detection by MS have not been described for these drugs. We are currently recruiting other patients to validate the identified VIP in an independent population. If confirmed, we will analyze the VIP by MS^2^ acquisition [36] in order to suggest identification for these putative biomarkers. These findings may help to (1) discuss the metabolic pathways involved in these clinical situations; and (2) to accurately assess the relationship between these compounds and medical therapies, using the appropriate website (http://www.drugbank.ca/). 

Despite the different types of shock or clinical characteristics of enrolled patients, PCA analysis revealed a correct homogeneity within each group of patients. Interestingly, we noted that 3 subjects were misclassified in the OPLS-DA score plot. We did not find any explanation after reading the medical history of these patients, except that the patient GJ had an inflammatory disease with an acute episode 10 days before the inclusion in this study. Thus, the metabolome profile may be informative in detecting atypical phenotypes. Pregnant women were included but we did not detect a specific profile of these subjects. Although some studies showed modification in global metabolism during pregnancy [37], few studies focused on the EBC of these subjects. Some authors highlighted a higher EBC pH in healthy pregnant women than that from healthy non-pregnant women [38]. These findings may have an impact on metabolic profile and need to be fully explored. We have to take into account that OPLS-DA provides optimistic results and building robust models to perform prediction requires a specific methodology. Importantly, external validation may be the most important criteria for considering a molecule as a true biomarker. The analysis on two sets of data (i.e., a training and validation sets) within a cohort appears insufficient; results need to be confirmed by an independent laboratory. Consensus on what is needed to validate a biomarker is missing both for targeted and untargeted studies. One of the crucial steps is the complete validation of the analytical method. Indeed, analytical techniques should be validated as for all biological parameters used in routine practice [39]. Establishment of guidelines could help to reduce spurious positive associations between diseases and some molecules.

To date we cannot discuss the potential pathophysiological ways involved in shock as the identification of VIP has not been finished yet. However, our data may be innovative as many studies are exclusively focused on immunological biomarkers. Numerous immune cell-associated surfaces and several soluble biomarkers have been described. Only some of them have been confirmed in septic patients [40]. For example, CD23, CD95, and CD80 expression on B cells have been identified as biomarkers in sepsis prognosis [41]. The most robust biomarkers of sepsis have been identified in blood by targeted analysis, such as the following routine biochemical parameters: creatinine, procalcitonin, bilirubin, and lactate concentrations; and hematological parameters [42]. To date, sensitivity and specificity of the available biomarkers are insufficient and new technologies or microbiologic assays are necessary to complete the exploration. As no single laboratory test is able to accurately diagnose sepsis, innovative techniques are necessary to make significant progress in this field. The aim of the present project was to develop the protocol for EBC collection and to validate the proof of concept in the interest of exploring metabolome in exhaled breath to find biomarkers. As these preliminary findings are promising, we may expect advances in biomarkers discovery based on metabolomics studies of this biological fluid [43].

We need further data with larger homogeneous populations, and we need to evaluate the specificity of theses markers and evaluate the relevance of this approach compared to other biological and/or clinical data. The concomitant analysis of blood metabolome in these patients may be very informative in order to provide complementary findings on pathophysiological methods.

## 5. Conclusions

To conclude, we performed a promising pilot study to evaluate the metabolome of exhaled breath condensation from patients of ICU. We correctly distinguished these patients, thus opening the way for further targeted metabolomics research. Moreover, a new independent study to validate our findings may enable the characterization of endophenotypes of patients, and may improve our knowledge of shock pathogenesis.

## Figures and Tables

**Table 1:** Characteristics of patients, NA: patients had not arterial gasometry during surgery; PEEP : Positive End Expiratory Pressure.

	Patients (*n* = 14)	Controls (*n* = 12)	*p*-Value
male	13	6	0.03
age	62 [49–83]	71 [53–89]	0.22
**Respiratory parameters**			
PEEP (cmH_2_O)	5 [0–8]	5.5 [5–8]	0.018
Volume (L/min)	6.1 [4–7.6]	9.2 [6.9–11.6]	0.000914
Fi O_2_ (%)	60 [50–60]	50 [33–80]	0.04
PaFi (mmHg)	NA	232 [126–415]	
ventilation duration (h)	0.75 [0.3–2]	20 [12–384]	0.0001
plateau pressure (cmH_2_O)	16.5 [14–120]	18 [16–28]	0.105
**Temperature (°C)**			
patient	36 [35.7–36.6]	37.9 [36.3–39.9]	0.00003
room	20	25 [24–26]	0.00012
